# Mesenteric Castleman Disease Mimicking Neuroendocrine Tumor

**DOI:** 10.7759/cureus.61549

**Published:** 2024-06-02

**Authors:** Manjesh K A, Sreedhar Rao Kota, Narender Mudigonda, Gokul Kumar, Kishore Abuji

**Affiliations:** 1 General Surgery, ESIC (Employee's State Insurance Corporation) Medical College, Hyderabad, IND; 2 General Surgery, ESIC (Employee's State Insurance Corporation) Hospital, Hyderabad, IND

**Keywords:** unicentric castleman disease, mesenteric mass, mesenteric lymph node, neuroendocrine tumour, castleman disease

## Abstract

Castleman disease (CD) is a benign lymphoproliferative disorder of unknown etiology, which can involve any part of the body. CD can involve a single lymph node (unicentric) or multiple lymph nodes (multicentric) of which unicentric CD is the most common type. The unicentric CD is usually localized, asymptomatic, and often appears as an incidental mass on radiographs, whereas multicentric CD is characterized by systemic involvement. Mesenteric involvement of CD is very rare. In this article, we present a case of the unicentric CD of small bowel mesentery, which mimicked a neuroendocrine tumor preoperatively.

## Introduction

Castleman disease (CD) is a lymphoproliferative disorder, which is non-malignant and rare in occurrence, arising from an unknown etiology. It can involve single (unicentric) or multiple (multicentric) lymph nodes in any part of the body [1]. The CD is further classified into two subdivisions: unicentric and multicentric, of which unicentric is the most common and usually follows an asymptomatic course. CD most commonly involves the mediastinum (70%) and other sites, which are the neck, axilla, retroperitoneum, pelvis, inguinal region, and pancreas [2]. We present a case of unicentric CD, which mimicked a neuroendocrine tumor preoperatively.

## Case presentation

**Figure 1:**
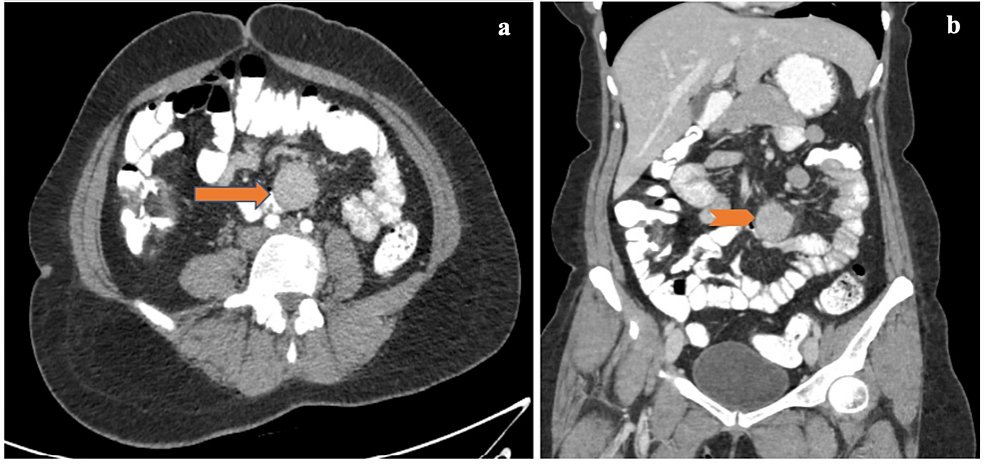
(a) Axial image of contrast-enhanced computed tomography showing homogenously enhanced lesion (solid arrow) in small bowel mesentery. (b) Coronal imaging showing homogenous lesion (solid chevron) at distal ileal mesentery.

A 36-year-old female underwent contrast-enhanced computed tomography (CECT) of the abdomen for liver donation for her child who was suffering from a genetic disorder of copper metabolism. Incidentally, the CECT showed soft tissue mass in the mesentery. Clinically, she was asymptomatic, but upon abdominal examination, the abdomen was soft, with no tenderness and no palpable mass. CECT of the abdomen revealed a well-defined, homogenously enhancing soft tissue density lesion measuring 2.5 x 3.2 cm, which was seen anterior to aortic bifurcation and left common iliac artery in the mesentery. The lesion shows bright arterial phase homogenous enhancement with contrast retention in subsequent phases, leading to a provisional diagnosis of the lesion as a neuroendocrine tumor (Figure 1).

Biochemical parameters including serum chromogranin A and urinary 5-hydroxyindole acetic acid (5-HIAA) were normal. The patient was screened for HIV and other virological investigations like hepatitis B surface antigen (HBsAg) and hepatitis C virus (HCV) serology, which are negative. To evaluate other lesions in the body, subsequently, she underwent Ga-68 DOTATATE PET-CT, which did not show any other lesions. After all investigations, a provisional diagnosis was made of a mesenteric neuroendocrine tumor. Another differential diagnosis is mesenteric gastrointestinal stromal tumors (GIST).

**Figure 2:**
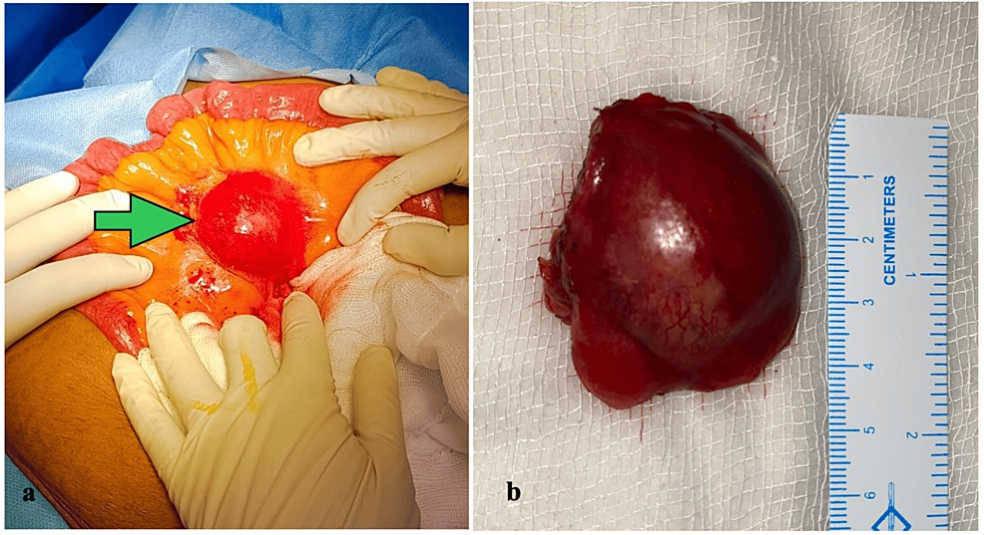
(a) Intraoperative image showing capsulated soft tissue lesion at distal ileal mesentery (solid arrow). (b) Resected specimen measuring 5 x 4 x 4 cm lesion.

After obtaining consent, the patient was prepared for surgery, and on mini-laparotomy and exploration, there was about 5 x 4 x 4 cm lesion arising from the distal ileal mesentery (Figure 2).

**Figure 3:**
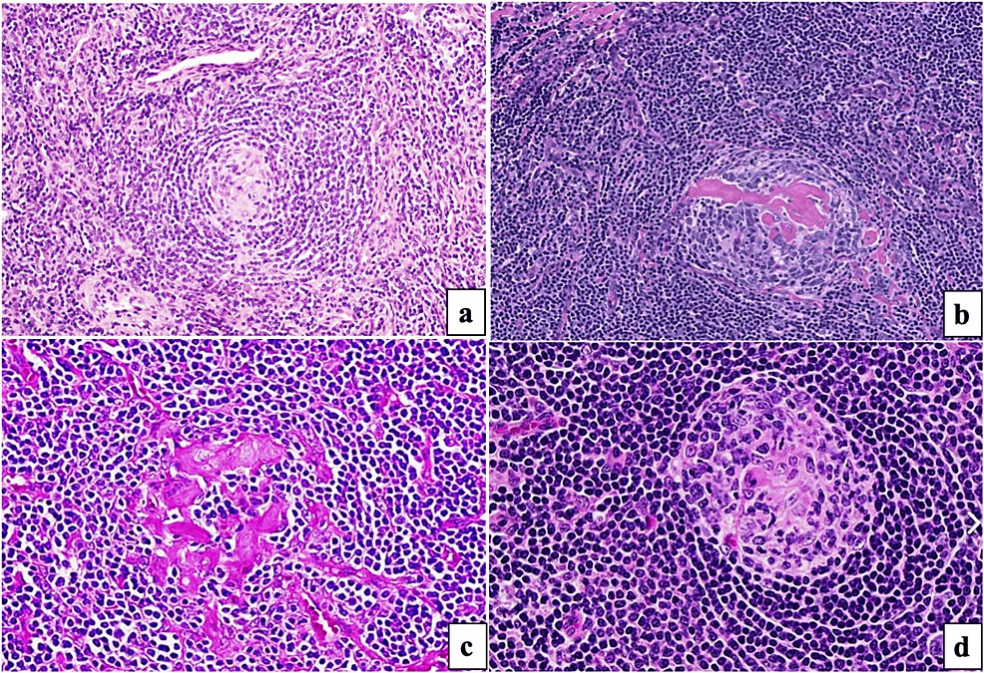
(a) Low magnification of the mesenteric lymph node showing thickened mantle zones (hematoxylin and eosin, H&E stain). (b) Sclerosed vessel in germinal center (H&E, 40x). (c) H&E stain, 100x magnification showing hyalinized vessels. (d) High magnification showing atretic germinal center (H&E stain).

Excision of the lesion with a 1-cm margin was done without sacrificing the bowel. On gross examination, a single gray-yellow to hemorrhagic soft tissue measuring 5 x 4.5 x 4.5 cm with a smooth surface capsulated was observed. On microscopic examination, the mesenteric mass was composed of lymph node architecture with the fibrocartilaginous capsule. The subcapsule shows numerous follicles with dilated, congested, and hyalinized blood vessels, and inter-follicular areas show diffuse sheets of mature lymphocytes (Figure 3).

The final diagnosis of mesenteric unicentric CD was made. The postoperative course was uneventful, and the patient was discharged on postoperative day 1 in stable condition. At six months follow-up, the patient was doing well and was under regular follow-up.

## Discussion

Benjamin Castleman described CD in 1956 after describing 13 patients with thymoma-like masses in the anterior mediastinum [3]. CD is a benign proliferative disorder of lymph nodes; the etiopathogenesis was still unknown, and some of the possible mechanisms include low-grade inflammation, autoimmunity, and immunodeficiency state. Multiple inflammatory mediators involved in the disease process, particularly IL-6 leading to neo-angiogenesis, have an important role in the pathogenesis of CD identified in preclinical animal models [4,5].

Clinically, CD is divided into unicentric and multicentric. Unicentric CD (UCD) is the most common (90%) type and often appears as an incidental mass on radiographs. Clinically, the usual presentation is a slow-growing asymptomatic solitary mass, and a few patients (10%) experience fever, weight loss, weakness, and symptoms due to the pressure effect of the mass [2]. In case of an abdominal lesion, the patient might present with compression of adjacent organs such as vomiting, postprandial discomfort, and abdominal or lumbar pain [6]. Multicentric CD (MCD) is less common and clinically symptomatic, characterized by systemic involvement, which presents as anemia, fever, increased C-reactive protein, ESR, and hypergammaglobulinemia [7].

Histologically, CD can be classified into hyaline vascular (HV) and plasma cell (PC) type [8]. HV type consists of lymphoid follicles with hyalinized blood vessels within it. HV type constitutes 80%-90% of CD and appears more commonly as UCD. PC type is composed of a spreading arrangement of plasma cells in the involved lymph nodes, and it is less common and appears usually as MCD [1].

The CD is commonly seen in the third and fourth decades of life with a median age of 35 years [9]. UCD is the most common type and frequently seen in mediastinum. The involvement of UCD in the abdomen and pelvis is about 10% in which mesenteric occurrence is very rare [10]. In our case, the patient has UCD in the distal ileal mesentery. Preoperative diagnosis of CD is very difficult; radiologically, CD findings are non-specific, and CT may show defined soft tissue density with homogenous enhancement with contrast. In our case also, it was provisionally diagnosed as NET of the mesentery, which is difficult to diagnose on the preoperative image. The HV type may show more contrast uptake than the PC type [11]. CT without histopathological reports will not give a definite diagnosis. The laboratory evaluation should include immunodeficiency and virological screening to rule out associated pathology. CD is a benign lymphoproliferative disorder that can be mistaken for lymphoma or other solid tumors. It is often associated with systemic autoimmune conditions such as Kaposi sarcoma and follicular dendritic cell (FDC) tumors. The HV type of CD is particularly linked to FDC tumors. Additionally, tumors like Hodgkin lymphoma and angioimmunoblastic T-cell lymphoma are known to mimic CD [12].

The curative treatment in all cases of UCD includes complete surgical resection; if not possible, partial resection is also preferred as it has a very low recurrence rate. Aggressive or extended surgical resection is not recommended as it might increase morbidity and mortality. Neoadjuvant rituximab and neoadjuvant radiotherapy are recommended in unresectable cases, which will cause tumor shrinkage and decreased vascularity so that the tumor can be resected with a low morbidity rate. CD can also be treated with radiotherapy/chemoradiotherapy, steroids, and/or immunotherapy (interferon-alpha and anti-IL-6 Abs), but these are not curative options [13]. Here, we reviewed the literature on some unicentric mesenteric CD with different surgical approaches (Table 1).

**Table 1:** Review of literature on some unicentric mesenteric Castleman diseases with different surgical approaches GERD: Gastroesophageal reflux disease.

Study	Age (years)/Sex	Presentation	Type of surgery	Intra-op findings	Histopathology
Kadoura et al., 2021 [14]	38/F	Abdominal discomfort	Complete excision, bowel not sacrificed	11.5 x 8.5 x 9 cm lesion occupied in mesentery	CD with hyaline vascular type
Kim et al., 2005 [15]	13/F	Abdominal al pain	Complete excision, bowel not sacrificed	5 x 4.5 x 4 cm, route of small bowel mesentery	CD with hyaline vascular type
Lv et al., 2015 [10]	71/F	Asymptomatic, incidentally found	Pancreas preserving segmental duodenectomy including lesion with duodenojejunal anastomosis (side-to-side)	4 x 4 x 3.5 cm occupied mesentery of duodenojejunal junction	CD with hyaline vascular
Bradai et al., 2021 [16]	62/F	Abdominal pain	Complete excision, bowel not sacrificed	3.5 x 2.5 x 1.5 cm lesion in mesentery	CD with hyaline vascular
El Demellawy et al., 2009 [17]	33/F	Recurrent symptoms of small bowel obstruction (intussusception)	NA	NA	CD with hyaline vascular
Bhogal et al., 2019 [1]	43/F	During GERD evaluation	Complete excision with bowel resection and anastomosis	3 cm mass in proximal small bowel mesentery	CD with hyaline vascular type
Bracale et al., 2017 [18]	33/F	Abdominal lump	Laparoscopic-assisted complete excision, bowel not sacrificed	9 x 8 x 4 cm in transverse mesocolon mesentery	CD with hyaline vascular type
Ozsoy et al., 2018 [2]	55/F	Abdominal pain	Complete excision along with bowel resection and anastomosis	6.7 x 6 x 5.5 cm ileal mesentery	CD with hyaline vascular type
Boovalli et al., 2014 [19]	39/F	Abdominal pain	Complete excision, bowel not sacrificed	8 x 7 x 5 cm	CD with hyaline vascular
Nayak et al., 2013 [20]	30/F	Abdominal pain with a lump	Complete excision, bowel not sacrificed	8 x 6 cm	CD with plasma variant

## Conclusions

The mesenteric CD is very rare, and accurate diagnosis through comprehensive evaluation, including histopathology, is crucial. Surgical resection remains the mainstay for unicentric CD, emphasizing the need for a multidisciplinary approach in managing this rare lymphoproliferative disorder.
